# The Challenges of Home Enteral Tube Feeding: A Global Perspective

**DOI:** 10.3390/nu7042524

**Published:** 2015-04-08

**Authors:** Omorogieva Ojo

**Affiliations:** Faculty of Education and Health,University of Greenwich, Avery Hill Campus, Avery Hill Road, London SE9 2UG, UK; E-Mail: o.ojo@greenwich.ac.uk; Tel.: +44-(0)20-8331-8626; Fax: +44(0)20-8331-8060

**Keywords:** enteral tube feeding, community, global perspective, developing countries, developed countries, home enteral nutrition

## Abstract

The aim of this review is to provide a global perspective of Home Enteral Tube Feeding (HETF) and to outline some of the challenges of home enteral nutrition (HEN) provisions. It is well established that the number of patients on HETF is on the increase worldwide due to advances in technology, development of percutaneous endoscopic gastrostomy techniques, and the shift in care provisions from acute to community settings. While the significance of home enteral nutrition in meeting the nutritional requirements of patients with poor swallowing reflexes and those with poor nutritional status is not in doubt, differences exist in terms of funding, standards, management approaches and the level of infrastructural development across the world. Strategies for alleviating some of the challenges militating against the effective delivery of HETF including the development of national and international standards, guidelines and policies for HETF, increased awareness and funding by government at all levels were discussed. Others, including development of HEN services, which should create the enabling environment for multidisciplinary team work, clinical audit and research, recruitment and retention of specialist staff, and improvement in patient outcomes have been outlined. However, more research is required to fully establish the cost effectiveness of the HEN service especially in developing countries and to compare the organization of HEN service between developing and developed countries.

## 1. Introduction

This article aims to provide an overview of the worldwide perspective of home enteral tube feeding (HETF) and outline some of the challenges of home enteral nutrition (HEN) provisions. Enteral tube feeding is an effective method of providing nutrients for individuals who are unable to meet their nutritional requirements in different healthcare settings across the world [1,2,3]. In a study by Klek *et al.* [4] in Poland, implementation of HEN improved clinical outcomes and decreased health care costs through weight gain in patients, reduced incidence of infectious complications and the number of hospital admissions. In another study in Malawi, Brewster *et al.* [5] reported that routine tube-feeding was associated with improved body weight gain in the treatment of kwashiorkor (protein deficiency). A nutritional support team in Nigeria has used high calorie enteral feed in the management of protein energy malnutrition in children [6]. On the other hand, HEN has been shown to improve the nutritional status and quality of life in patients with advanced gastric cancer in China [7].

The use of HETF has become more common globally due to advances in technology, development of percutaneous endoscopic gastrostomy (PEG) technique and governments’ policy of shifting healthcare provisions from costly acute hospitals to community settings [8,9,10,11]. These have also ensured that more individuals on enteral feeds now live in the community. In the UK, a 42.78% increase over a 10-year period in patients receiving HEN has been reported [12], although there is evidence of a yearly increase (20%–25%) in the number of people on enteral feed [13]. According to Moreno Villares [14], home enteral nutrition was developed after home parenteral nutrition, however, it has grown rapidly in some countries. There are significant international differences in prevalence and growth rate of home enteral nutrition, in the organisation of nutritional support services, and in financial arrangements [15]. Although the incidence of HETF may be difficult to determine, estimates of 460 (United States) and 40 (Spain) patients per million inhabitants have been reported [14]. In most countries around the world, neurological diseases, such as cerebrovascular accident, multiple sclerosis, and cancer especially, head and neck cancer, are the most frequent indications for HETF [12,14].

In a previous study by Ojo [12] in the UK, 187 out of the 257 patients on HETF, representing 72.76% were receiving enteral nutrition through percutaneous endoscopic gastrostomy. This value appears similar in other parts of Europe, although this only occurs in 25% of cases in Spain [14].

Despite worldwide application of HETF, significant differences remain between developed and developing countries in terms of the challenges, especially with respect to funding, organisation, infrastructure, procurement of feeds, pump, ancillary items, research and development, and management of patients and related complications.

### Organisation of Home Enteral Tube Feeding

Irrespective of the strategy for delivering HEN service, the procurement and supply of enteral feeding pump, feed and ancillary items require an effective process in order to ensure a seamless delivery and promotion of patients’ outcomes. This is essential because there should be continuity of service provision and enteral nutrition management when patients are discharged home from hospital. In the USA and UK, national standards require patients on HETF to be well trained, to have written protocols for trouble-shooting and for undertaking routine procedures, and to have 24-h telephone contacts in cases of emergency. While these have been developed in the US and UK, other countries appear to have local standards [15].

Furthermore, in the USA, the role of companies in the supply and delivery of pumps, feed, ancillary items, management of patients on HEN and associated complications is quite significant while in Europe, HEN is usually coordinated from regional or district hospitals [15]. Other approaches to HEN in the USA have been outlined by Newton and Barnadas [3], who reported that home enteral nutrition is often started without the usual resources of the hospital. In addition, dieticians working in home care mostly assume primary role in patient care and management, and have to have knowledge and expertise with the wide variety of enteral equipment and access devices.

In Poland, there is evidence that HEN includes complex monitoring by a nutrition support team [4]. With respect to the UK, enteral feed, feeding accessories and pump are supplied to patients in the community through different strategies and the management could be one or a combination of the following; self care, carers, care home registered nurses, general practitioner (GP), Home enteral feeding service provided by a dietician, community nursing service, Home care company and the HEN team [16].

However, two of the most common methods of supplying patients with enteral feed, feeding accessories and pump in the UK are Hospital Enteral Nutrition Service and Community or Home Enteral Nutrition (HEN) Service.

Under the Hospital Enteral Nutrition service, the nutrition department or the nutrition team based in hospital is responsible for the care and management of patients on HEN including the supply of feed, feeding accessories and pump in hospital and home. The nutrition support team in hospital often provides home visits or outpatient clinics and for those patients who have been discharged home from hospital, the continued supply of feed and feeding accessories is usually by means of prescription using the FP10 (prescription form) [16,17]. The delivery of the feeding pumps to HEN patients is often through the community nursing. National Health Service (NHS) supplies organisations, or the receive the equipment directly from the manufacturer and costs are charged back to the hospital budget [18].

In contrast, the UK Community or Home Enteral Nutrition Service is usually multidisciplinary team that consists of healthcare professionals and support staff who have diverse clinical qualifications, skills, knowledge and experience [19]. These specialist healthcare professionals include the nutrition nurse specialist, dietician, and speech and language therapist. One of the primary features of the HEN service is that patients’ feeds are managed “off script” and therefore do not require the use of FP10 [20]. The HEN service is the model of choice and requires a clear pathway between acute and community care and other partners including companies in order to ensure an effective delivery system [8,21,22]. In a study that compared hospital enteral nutrition and home enteral nutrition in China, Wang *et al.* [23] reported that patients with intestinal fistulae in the HEN group had shorter hospital stay than patients in the hospital group. Furthermore, the cost of treatment was significantly lower and the quality of life significantly improved in the HEN group compared with the Hospital enteral nutrition group. In studies from a number of countries around the world reviewed by Majka *et al.* [24], multiple intervention strategies were adopted in the management of enteral tube feeding.

Based on the above, the objective of this review is as follows;

- To explore the issues around home enteral tube feeding in providing nutritional support globally.

The research question is:

- Are there challenges in the use of home enteral tube feeding as a global strategy in nutritional support provision?

## 2. Method

This review involved a literature search of articles on challenges of home enteral nutrition across the globe. It included a general scoping of the databases, which found one systematic review on international perspective on artificial nutritional support in the community published in 1995 by Elia [15]. This review based its findings on evidence drawn from studies relating to both Home parenteral and enteral nutrition. Since the publication, a number of other studies have been published which form the basis for the current review.

The main data bases searched included EBSCO Host/Health Sciences Research databases (encompassing Academic search premier, Medline, Psychology and Behavioural sciences collection, PSYCINFO, SPORTDISCUSS and Cumulative Index to Nursing and Allied Literature (CINAHL) Plus, PubMed and SwetsWise. The search terms included; challenges of enteral nutrition, challenges “and” home enteral tube feeding, enteral nutrition “and” Europe, enteral nutrition “and” Africa, enteral nutrition “and” South America, enteral nutrition “and” Asia, enteral nutrition “and” central America, enteral nutrition “and” America, enteral nutrition “and” Australia.

### 2.1. Inclusion and Exclusion Criteria

Searches included articles published between 1995 and 2014 covering the areas of interest except, one study that was published in 1988 and was included due to the limited studies published on Africa. Only studies written in English language were included and those that did not meet the above inclusion criteria were excluded from the current review.

### 2.2. Data Analysis

Based on the criteria outlined for exclusion and inclusion, eight studies on enteral nutrition complications (Table 1) were selected. Seven studies on cost effectiveness of enteral nutrition (Table 2) were also included although some of these studies were previously cited in Table 1. Most of these studies were conducted in Europe, North America and Australia. While studies carried out in developing countries in these aspects of enteral nutrition were limited, there were studies from around the world that provided an overview of the challenges of enteral nutrition, which were included.

## 3. Results

With respect to enteral nutrition complications, the results show that across the globe, the main sources of enteral nutrition complications are due to stoma site infection, tube dislodgement, tube blockage, tube leakage, diarrhoea, overgranulation, vomiting, and pneumonia (Table 1).

While formation of overgranulation tissue (67%) was the highest form of complication in Canada [25], constipation (48%) was the highest in Ireland [26]. In Greece, inadvertent removal of tube (45.1%) was highest and in Australia insertion site infection was 41% [27,28]. In Turkey, while tube displacement was highest at 7.6%, wound infection was 3.3% [29]. Overgranulation of PEG site was 26.7% compared with 6.7% for balloon gastrostomy site in the UK, while pneumonia was the most frequent form of complication at 55.9% in Brazil [1,30].

With respect to the findings on the cost effectiveness of enteral nutrition, one study showed variation in the daily overall costs of HEN from 7 to 25 Euros across Europe [31] (Table 2). On the other hand, studies in Poland and the US showed significant improvements in patient outcomes and cost of hospitalisation following implementation of HEN and nutrition support clinic [4,32].

One study in the US revealed that HEN is significantly cheaper compared with Home parenteral nutrition [33]. The Australian study showed that inpatient cost of PEG patients was significantly more than for patients on nasogastric feeding tube (NGT) [28]. The UK study found that the cost effectiveness of enteral tube feeding, where non-medical costs are paid privately, compares favourably with other interventions, but it was not so when the state pays all non-medical costs [34]. The study which compared the impact of home and hospital enteral nutrition in China found that the HEN group had shorter hospital stay, significant reduction in cost of treatment, improved quality of life although no significant difference in the incidence of complications [23].

## 4. Discussion

### 4.1. Challenges of Current Organisation of Home Enteral Tube Feeding

In developed countries of Europe and the USA, where organised HEN services exist, challenges in the areas of funding, the number of HEN services, research and development, multidisciplinary team working, managing the HEN service including problems with delivering enteral feed and accessories and complications due to pump, feed and stoma site are often the main issues of concern [1,12,27,32,35,36]. Details of these difficulties of the HEN service globally have been fully outlined in Table 1. For example, in the UK, many National Health Service (NHS) Trusts do not have a HEN team and this will limit their capacity for service improvement and delivery [19].

Funding is a major challenge in the management of HETF anywhere in the world and with the on-going economic crisis influencing health care, its cost-effectiveness has been questioned [4]. British Artificial Nutrition Survey (BANS) reports that 40% of centres have no budget allocated specifically for enteral nutrition support [10].

According to Elia [15], the use of home enteral nutrition in different countries appears to be generally related to the overall expenditure on health (government and private), and percentage of the gross domestic product (GDP) expended on health. In Asian countries such as India and Pakistan, and in many parts of Africa, where the figure is less than 4%–5% of the GDP, home enteral nutrition is not common [15]. By contrast, in the USA where the GDP expended on health from private expenditure alone was 11.7% in 1991, home enteral nutrition is used more than anywhere else in the world [15]. This is followed closely by Western European countries where expenditure on health is usually 6%–9% of GDP although estimates from eastern European countries with limited information is likely to be low [15]. For example, in the UK based hospital enteral nutrition service, the effective management of feed, pump and ancillary items, and patients on HEN may be affected because there is no dedicated team in the community to follow up in the care and maintenance of the service. Under this method, the unit cost of feed is much higher because individual patient in the community rely on the use of FP10 (prescription form) to procure their feed compared to when feeds and feeding accessories are purchased in larger quantities [18]. In addition, due to conflicting pressures dietetic support may not be routinely available for patients on HETF, even on outpatient basis.

Although the community home enteral nutrition service purchases feed “off script” and thus can save money from the bulk purchase of feeds, challenge of multidisciplinary team working can militate against effective service delivery. For example, although the roles and responsibilities of the different HCPs are distinct, there are areas of overlap, which may become sources of friction. The number of different HCPs working within the HEN team who visit the patients regularly for assessment and reviews can become blurry and confusing to the patients due to their number and different times of visits [19].

Due to the specialist nature of the HEN service, recruitment and retention of qualified staff are some of the difficulties providers have to address. Therefore, it is not uncommon to find some HEN services having developmental roles such as Nutrition Support Nurse, Nutrition Nurse Specialist working alongside the Clinical Lead Nutrition Nurse Specialist [8].

Despite the worldwide recognition of HETF as a useful method of meeting the nutritional requirements of patients and as a life-saving procedure, difficulties often arise during and post tube insertion. These problems appear to the similar globally although the levels of prevalence may vary [25,26,28,29,30,37] (Table 1).

Other possible challenges confronting the HEN service include the supply of feed, pump and ancillary items. Evans *et al.* [38] reported that 20% of patients receiving HEN were not contacted until seven days or more after discharge in the UK. In addition, 47% of patients did not receive a delivery until seven or more days after discharge, while 41% reported missing equipment from their first delivery. In the same study, 17% of patients reported difficulty getting their GP to write a prescription. Those patients whose deliveries were not organised directly with the home delivery company by the hospital were more likely to have delayed delivery of seven or more days after discharge (63% compared with 21% of those directly organised by regional hospital) [38].

**Table 1:** Incidence of Enteral Feeding Complications.

Citation	Country of Study	Type of Study	Number of Patients	Outcomes
Hall *et al.*, 2014 [32]	USA	Retrospective quality analysis	52 patients	- Approximately 30% of patients seen at least once for clogged tube and 43.3% for tube leakage. - One patient required a procedure for tube re-insertion
Corry *et al.*, 2009 [28]	Australia	Prospective study to compare PEG tubes and NGT tubes in terms of nutritional outcomes	32 PEG and 73 nasogastric tube (NGT) patients	- PEG patients had significantly less weight loss at 6 weeks post treatment (median 0.8 kg gain *versus* 3.7 kg loss), but had a higher insertion site infection rate (41%). - There was 62% tube dislodgement in the NGT group compared with 19% for PEG group
Alivizatos *et al.*, 2012 [27]	Greece	Retrospective review of medical records	31	Accidental removal of tube (broken tube, plugged tube; 45.1%), tube leakage (6.4%), dermatitis of the stoma (6.4%), diarrhoea (6.4%)
McNamara *et al.*, 2000 [26]	Ireland	Retrospective survey	50	Blocked tube (30%), local infection at stoma site (16%), tube replacement (26%), diarrhoea (34%), vomiting (40%), constipation (48%).
Crosby and Duerksen, 2005 [25]	Canada	Retrospective Survey	55 out of 221 patients completed the survey	Granulation tissue formation (67%), broken or leaking tube (56%), leakage around the tube site (60%), stoma infection requiring antibiotics (45%)
Martins *et al.*, 2012 [30]	Brazil	-	79 patients	- 91.2% presented some complications such as pneumonia, catheter loss, diarrhoea, constipation, fluid leakage, tube obstruction, reflux. - Pneumonia was the most frequent complication, occurring in 55.9% of cases
Ojo, 2011 [1]	UK	Retrospective review	30 patients	Overgranulation of stoma site (PEG, 26.7%; Balloon gastrostomy tube, 6.7%), infected stoma site (PEG, 6.7%; Balloon gastrostomy tube, 13.3%) during initial visit.
Erdil *et al.*, 2005 [29]	Turkey	-	85 patients	More than 30 days after insertion of PEG; Tube occlusion (4.3%), tube displacement (7.6%), wound infection (3.3%), peristomal leakage (2.2%), reflux and vomiting (1.1%), peritonitis (1.1%).

**Table 2:** The Cost—effectiveness of Enteral Nutrition.

Citation	Country of Study	Type of Study	Number of Patients	Outcomes
Klek *et al.*, 2014 [4]	Poland	Observational multicentre study	456 HEN patients	- HEN enabled weight gain, stabilised liver function. - HEN implementation reduced incidence of infectious complications (37.4% compared with 14.9%), the number of hospital admissions [1.98 ± 2.42 (mean ± SD)] before and 1.26 ± 2.18 after enteral nutrition. - The length of hospital stay was 39.7 ± 71.9 compared with 11.9 ± 28.8 days. - The mean annual costs ($) of hospitalisation were reduced from 6500.20 ± 10,402.69 to 2072.58 ± 5497.00
Reddy, 1998 [33]	USA	Retrospective Review	-	For Home parenteral and Home enteral nutrition respectively; Annual cost per patient solution ($55,193 ± 30,596; 9605 ± 9327) (mean ± SD), annual cost of hospitalisation (0–$140,220; 0–$39,204), Annual number of hospitalisations per patient (0.52–1.10; 0–0.5), Health status (significantly lower; significantly higher).
Hall *et al.*, 2014 [32]	USA	A retrospective quality analysis	52 patients	Complications and high cost interventions, including emergency room visits, hospital admissions and surgical tube re-insertions were significantly reduced after implementation of nutrition support clinic.
Elia and Stratton, 2008 [34]	UK	Cost-utility analysis	-	- The cost effectiveness of enteral tube feeding in patients with cerebral vascular accident receiving enteral tube feeding at home or nursing homes, where the non-medical costs are paid privately compared favourably with other interventions. - The cost effectiveness of enteral tube feeding in nursing homes when the state pays all non-medical costs compared unfavourably with other treatments.
Corry *et al.*, 2009 [28]	Australia	Prospective study to compare PEG tubes and NGT tubes in terms of nutritional outcomes	32 PEG and 73 NGT patients	- The median nights stay in hospital was 4 for the NGT patients compared with 14 for the PEG patients. - The inpatient cost for PEG patients would be $3556 *versus* $1016 (Australian dollars) for the NGT group.
Wang *et al.*, 2013 [23]	China	Comparative study between Home enteral nutrition and Hospital enteral nutrition	Home enteral nutrition =42; Hospital enteral nutrition = 40; Normal control = 40	- The HEN group had shorter hospital stay, significant reduction in cost of treatment and improved quality of life. - No significant difference in the incidence of complications
Hebuterne *et al.*, 2003 [31]	Belgium, Denmark, France, Germany, Italy, Poland, Spain, UK	A European Multicentre Survey	1397	- Daily costs of HEN were not available in centres from Denmark and the UK. - In other centres of Europe, the daily overall costs of HEN varied from 7 to 25 Euros. - These costs include costs of the formula, the infusion pump, micronutrients, bags, tubing and dressing and do not include cost of the care giver, cost of re-hospitalisation and medical monitoring.

### 4.2. Cost Effectiveness of Home Enteral Nutrition

The cost effectiveness of home enteral nutrition may be assessed based on the service being provided and strategies for delivering the service [28,31,32,34]. Details of the cost-effectiveness of the HEN service are clearly outlined in Table 2. In a systematic review and meta-analysis of studies across the world, Majka *et al.* [24] showed that 93.3% of the studies reviewed reported beneficial effects in terms of patients’ outcomes, staff outcomes and costs of care due to care coordination and/or team approach. These views are related to the findings of the current review which show that the HEN service provide significant improvement in patients’ outcomes [4,23,33].

Patient outcomes may be based on mortality, quality of life scores, infections and hospital admissions [28,31,32,34]. According to Majka *et al.* [24], the average hospital cost per patient was reduced by $623.08. In another review conducted by Michael *et al.* [39] in the USA, it was reported that the average savings from enteral nutrition due to reduced adverse event risks was about $1500 per patient and savings from reduced hospital length of stay was about $2500 per patient. It was noted that shifting 10% of adult patients in the USA managed parenterally to enteral nutrition would save $35 million annually due to reduced adverse events and another $57 million from shorter stays in hospital [39].

Pritchard *et al.* [40] reported that observational non-randomised studies that compared groups receiving parenteral and enteral nutrition found few differences in clinical outcomes between groups but showed lower costs in enteral nutrition patients. For example, while in 19 US patients about to have major abdominal surgery, mean daily costs for enteral nutrition were just under half the costs of parenteral nutrition of about $100 pay, in 24 UK patients undergoing orthotopic liver transplantation, there was a 10-fold difference (£7 per day compared with £75–85) [40].

Wilhelm *et al.* [41] carried out a theoretical cost analysis between inpatients and outpatients on percutaneous endoscopic gastrostomy in the US. According to the authors, the actual outpatient charge was $135 per patient compared to $1155 per patient for a theoretical admission. A potential cost savings of $29,120 in 26 out-patient procedures and a projected average cost savings per patient of $1020 were reported by Wilhelm *et al.* [41].

## 5. Strategies for Improving Home Enteral Tube Feeding

There is urgent need to develop the infrastructure for HEN service in developing countries in Africa, Asia and South America in order to meet the needs of the patients who may require these services. With respect to developed countries of Europe and North America, there is the need to improve cooperation and communication between nurses in hospitals and in communities, as well as for increasing nurses’ level of knowledge, to make home enteral tube feeding work in a safe way [42]. In addition, the roles and responsibilities should be clarified between the different healthcare professionals who should also understand guidelines for the care of tube feeding and discharge process [43,44,45]. There should also be clear pathways of referral and communication between acute, community, companies and patients.

The establishment of national regulations and legislation (or even regional regulations within a country) should be encouraged because they provide important advantages, including a uniform distribution of specialist HEN services across the country and clear financial guidelines, which should ensure that appropriate standards for selection and management are attained ensuring that government departments are fully aware of the issues involved [15].

In addition, it is essential to build trust between the different professions in the team, and support colleagues to take on new roles that are not normally associated with their traditional roles [26,46]. As part of the building of effective multidisciplinary HEN team, it will be useful to have regular training, away days, caseload review meetings and a team charter in place.

Creating the enabling environment for HCPs working within the HEN service to conduct research and clinical audits will no doubt provide the opportunity for service improvement and staff development. The issue of inappropriate placements of enteral feeding tubes and/or overprescription of enteral feed and ancillary items could have far reaching implications in terms of costs to the health service. A useful strategy could be to ensure appropriateness of indications of enteral nutrition provisions and the range of feeding tubes and accessories.

## 6. Conclusions

Home enteral tube feeding is a useful method of supporting the nutritional needs of patients in the community who are unable to meet their nutritional requirements through oral intake alone. Although its use has been globally acclaimed, differences exist in home enteral nutrition provisions across the world.

The factors militating against the effective delivery of HEN service include; funding, level of organisation, lack of national and international standards and infrastructure, problems of procurement of feeds, pump, ancillary items, research and development, and management of patients and associated complications including tube and stoma complications.

In order to address these problems, there has to be development of national and international standards, guidelines and policies for enteral nutrition provisions, increased awareness and funding by government at all levels, development of HEN services that will provide the enabling environment for multidisciplinary team work, clinical audit and research, recruitment and retention of specialist staff, and improvement in patient outcomes. However, more research is needed to fully establish the cost effectiveness of the HEN service especially in developing countries and to compare the prevalence of complications, cost implications and organization between developed and developing countries.
